# Aspirin decreases hepatocellular carcinoma risk in hepatitis C virus carriers: a nationwide cohort study

**DOI:** 10.1186/s12876-020-1158-y

**Published:** 2020-01-09

**Authors:** Yen-Hsiang Liao, Ren-Jun Hsu, Tzu-Hwei Wang, Chen-Ta Wu, Sheng-Yao Huang, Chung-Y. Hsu, Yuan-Chih Su, Wen-Lin Hsu, Dai-Wei Liu

**Affiliations:** 1 Department of Radiation Oncology, Hualien Tzu Chi Hospital, Buddhist Tzu Chi Medical Foundation, Hualien, Taiwan; 2 School of Medicine, Tzu Chi University, Hualien, Taiwan; 3 Graduate Institute of Clinical Medical Science, China Medical University, Taichung, Taiwan; 4 College of Medicine, China Medical University, Taichung, Taiwan; 5 Management Office for Health Data, China Medical University Hospital, Taichung, Taiwan

**Keywords:** Aspirin, Hepatitis C virus carrier, Hepatocellular carcinoma

## Abstract

**Background:**

Aspirin has been found to lower the occurrence rates of some cancers through the inhibition of the cyclooxygenase enzyme. For example, there is a well-known association between aspirin use and the occurrence of hepatocellular carcinoma (HCC) in hepatitis B virus (HBV) carriers. However, the association, if any, between aspirin use and HCC in hepatitis C virus (HCV) carriers is unknown. Therefore, this study compared the occurrence rates of HCC in HCV carriers treated with or without aspirin.

**Methods:**

The participants in this retrospective cohort study consisted of people newly diagnosed with HCV in Taiwan from 2000 to 2012. Those who were treated with aspirin were defined as the control group, whereas those not treated with aspirin were defined as the comparison cohort. We used a 1:1 propensity score matching by age, sex, comorbidities, drugs, diagnosis year, and index year with covariate assessment.

**Results:**

Our study sample consisted of 2980 aspirin-treated HCV carriers and 7771 non-aspirin-treated HCV carriers. After propensity score matching, each cohort consisted of 1911 HCV carriers. The adjusted hazard ratio (aHR) of HCC incidence in the aspirin users (aHR = 0.56, 95% CI = 0.43–0.72, *p < 0.001*) was significantly lower than that in the non-aspirin users. A Kaplan-Meier analysis showed that among the HCV carriers, the aspirin users had a lower cumulative incidence rate of HCC over the first 10 years of aspirin treatment (*p < 0.0001*).

**Conclusions:**

The HCC incidence rate was lower in the aspirin-using HCV carriers than in the non- aspirin-using HCV carriers, indicating that the effects of aspirin might occur through inhibition of the cyclooxygenase enzyme pathway. Moreover, protection from HCC was provided by less than a year of aspirin treatment, while treatment with aspirin for 1 to 2 years exhibited the greatest protective effect. We therefore encourage aspirin treatment to prevent HCC in HCV carriers.

## Background

Hepatocellular carcinoma (HCC) is one of the most common malignant neoplasms in the world. The incidence of HCC has increased not only in the United States [1] but also in Taiwan [2]. Hepatitis B virus (HBV) infections, hepatitis C virus (HCV) infections, and alcoholic liver cirrhosis are the main risk factors for HCC [3–5]. Chronic inflammation occurring through the COX-2 pathway is one of the most important process in the induction not only of liver cirrhosis but also of HCC [6].

Aspirin has been widely used as an analgesic and anti-inflammatory drug. It also plays an important role in preventing cerebrovascular and cardiovascular thrombosis and even lowering the associated mortality, especially in diabetes mellitus patients [7–10]. Aspirin may also prevent the occurrence of some cancers, such as colorectal cancer [11–14], lung cancer [15], prostate cancer [16], and head and neck cancer [17], as well as HCC [18]. Its effects are likely due to the inhibition of the cyclooxygenase enzyme. Aspirin, therefore, prevents carcinogenesis, cell invasion, angiogenesis, and metastasis through its promotion of prostanoid synthesis [19]. It also induces apoptosis by altering the Bax/Bcl-2 ratio and activating death receptors [19].

After being infected with HCV, a patient becomes an HCV carrier. Aspirin treatment can decrease the occurrence of HCC through the inhibition of the cyclooxygenase enzyme pathway. Some retrospective reports have shown that aspirin decreases the incidence of HCC [18, 20]. However, the association between aspirin use and the incidence of HCC has been evaluated only in HBV carriers, not in HCV carriers [21].

Therefore, we conducted a nationwide cohort study to evaluate the association between aspirin use and the incidence rate of HCC in HCV carriers.

## Methods

### Data sources

We used the Longitudinal Health Insurance database 2000 (LHID2000) to conduct this cohort study. The LHID2000 is a sub-dataset of Taiwan’s National Health Insurance Research Database (NHIRD) that contains 1 million random subjects. The NHIRD contains the medical care data from the National Health Insurance (NHI) program, which covers over 99% of the population of Taiwan. The NHIRD provides diagnosis data according to the International Classification of Diseases, 9th revision, Clinical Modification (ICD-9-CM), as well drug treatment and demographic information, with the identification of each individual included in the database being replaced by a random sequence. The Research Ethics Committee of China Medical University and Hospital in Taichung, Taiwan approved this study (CMUH104-REC2–115(CR-2)).

### Study population

The study population consisted of subjects who had been newly diagnosed with HCV (ICD-9-CM codes: 070.41, 070.44, 070.51, 070.54, and V02.62) from 2000 to 2012 and had at least two HCV outpatient records or one HCV inpatient record in the database. We then classified the study population of individuals diagnosed with HCV into aspirin users or non-aspirin users. The date on which aspirin was first received was defined as the index date. The exclusion criteria were as follows: (1) age less than 20 years, (2) HBV patient, (3) HIV infection, (4) treatment with an interferon or direct-acting anti-viral before the index date, and (5) a diagnosis of HCC before the index date. The aspirin users and non-aspirin users were matched using propensity score matching with gender, age, HCV diagnosis year, number of years of aspirin use, and interval between HCV diagnosis and aspirin use.

### Main outcome and confounding factors

The main outcome of this study was a new diagnosis of HCC (ICD-9-CM code: 155). Each case of HCC was identified according to the records of the registry for catastrophic illness patients. If another disease occurred before the index date, it was considered as baseline comorbidity. These comorbidities included hypertension (ICD-9-CM codes: 401–405), diabetes mellitus (ICD-9-CM code: 250), moderate or severe liver disease (ICD-9-CM codes: 456.0–456.2, 572.2–572.4, 572.8), myocardial infarction (ICD-9-CM codes: 410, 412), congestive heart failure (ICD-9-CM codes: 398.91, 402.01, 402.11, 402.91, 404.01, 404.03, 404.13, 404.91, 404.93, 425.4, 425.5, 425.7~425.9, 428), and ischemic stroke (ICD-9-CM codes: 433–436). The use of medications such as anti-hypertensive agents, hypoglycemic agents, coumadin and heparin, other antithrombotic agents, and non-steroidal anti-inflammatory drugs (NSAIDs) within 180 days before the index date was considered a confounding factor.

### Statistical analysis

We analyzed the HCC risk among the HCV carriers treated with aspirin and the HCV carriers not treated with aspirin. The follow-up period was the duration between the index date and the date of occurrence of the main outcome, withdrawal from the NHI program, death or the end of 2013, whichever came first. The crude, adjusted hazard ratios (aHRs) and corresponding 95% confidence intervals (95% CIs) were estimated using a Cox proportional hazards regression model without and with adjustment for gender, age, and confounding factors. We also assessed the aspirin effect among different strata of patient characteristics. We calculated the cumulative incidence of HCC using the Kaplan-Meier method and examined the difference in two trends using the log-rank test. All analyses were performed with the SAS statistical package, version 9.4 (SAS Institute, Cary NC). The significance level was set at 2-tail *p < 0.05*.

## Results

**Fig. 1:**
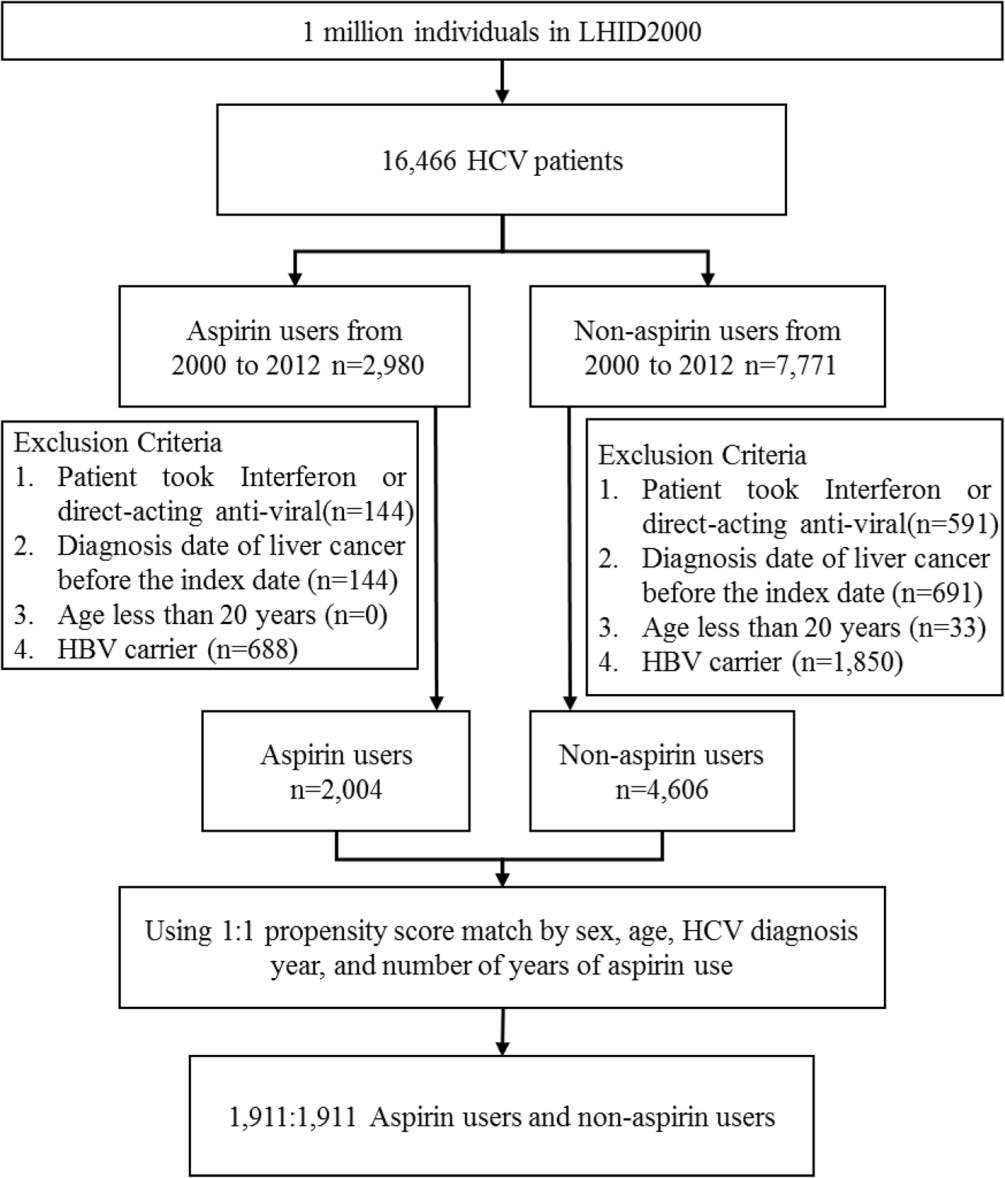
Flow diagram of the selection of study subjects

We documented 2980 aspirin users and 7771 non-aspirin users among the 16,466 individuals newly diagnosed as HCV carriers from 2000 to 2012 (Fig. 1). After applying the exclusion criteria to those patients, propensity score matching was applied to 2004 aspirin users and 4606 non-aspirin users. The final study population consisted of 1911 HCV carriers treated with aspirin and 1911 HCV carriers not treated with aspirin. Table 1 shows that there were no significant differences between these aspirin-treated and non-aspirin-treated groups in terms of their gender, age, and baseline comorbidities. However, the proportions of patients in the aspirin-treated group who used antihypertensive agents, coumadin and heparin, antithrombotic agents, and NSAIDs were significantly greater than the proportions of patients in the non-aspirin-treated group who did so, although there was no significant difference between the two groups in terms of the proportions who used hypoglycemic agents.

**Table 1:** Demographic characteristics and covariates in HCV patients (carriers) treated with and without aspirin

Variables	Aspirin				*p* value*
	No (*N* = 1911)		Yes (*N* = 1911)		
	n	%	n	%	
Gender					0.33
Female	1027	53.7	997	52.2	
Male	884	46.3	914	47.8	
Age, years					0.01
< 40	98	5.13	96	5.02	
40–59	606	31.7	580	30.4	
60–79	936	49.0	1025	53.6	
≥ 80	271	14.2	210	11.0	
Mean (SD)	64.6	14.4	64.3	13.5	0.58^†^
Baseline comorbidity					
Hypertension	1462	76.5	1410	73.8	0.05
Diabetes mellitus	905	47.4	848	44.4	0.06
Moderate or severe liver disease	34	1.78	23	1.2	0.14
Myocardial infarction	123	6.44	133	6.96	0.52
Congestive heart failure	431	22.6	429	22.5	0.94
Ischemic stroke	1245	65.2	1212	63.4	0.27
Drugs					
Anti-hypertensive agents	1003	52.5	1506	78.8	< 0.001
Hypoglycemic agents	114	5.97	141	7.38	0.08
Coumadin and heparin	55	2.88	175	9.16	< 0.001
Other antithrombotic agents	153	8.01	491	25.7	< 0.001
NSAIDs	931	48.7	1140	59.7	< 0.001

*Chi-square test; †Two sample t-test

Abbreviations: *SD* Standard deviation, *NSAIDs* Non-steroidal anti-inflammatory drugs

**Fig. 2:**
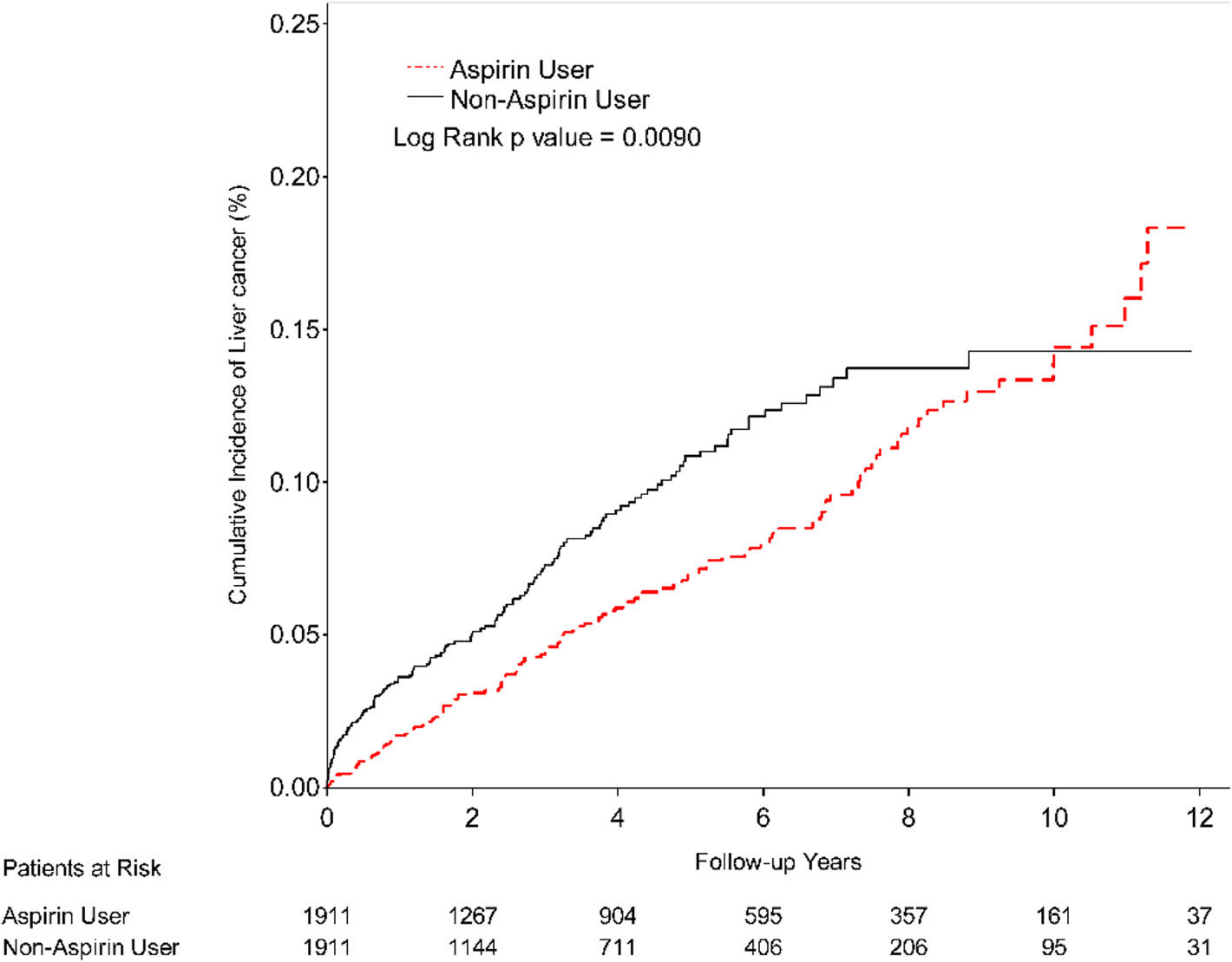
Kaplan-Meier curves showing the cumulative incidence of hepatocellular carcinoma (HCC) in hepatitis C virus carriers with or without aspirin treatment. Red line presents aspirin users. Black line presents non-aspirin users. X-axis presents follow-up years. Y-axis presents cumulative incidence of hepatocellular carcinoma

Compared with the non-aspirin users, the aspirin users had a lower risk of HCC (adjusted HR = 0.56, 95% CI = 0.43–0.72, *p < .001*; Table 2). The lower HCC risk in the aspirin users was further displayed by the cumulative HCC incidence trend (Fig. 2). Furthermore, Table 2 demonstrates a 1.71-fold higher HCC risk in the male patients than in the female patients (adjusted HR = 1.71, 95% CI = 1.35–2.18, *p < 0.001*). The HCC risk was also higher in those individuals who were older than 40 years than in those who were less than 40 years old. The comorbidities were not associated with the risk of HCC in the HCV carriers (that is, the risk of HCC in those individuals with a comorbidity was similar to that in those with no comorbidities). However, the adjusted HR in those who received anti-hypertensive agents was 1.94-fold higher than the adjusted HR in those who did not (95% CI = 1.45–2.6, *p < 0.001;* Table 2). Other medications were not associated with the risk of HCC.

**Table 2:** Cox model measured hazard ratios (HRs) and 95% confidence intervals of HCC occurrence associated with and without aspirin use and covariates among HCV carriers

Characteristics	Event no.	Crude			Adjusted		
	(*n* = 278)	HR	(95% CI)	*p* value	HR	(95% CI)	*p* value
Aspirin							
No	147	1	reference		1	reference	
Yes	131	0.73	(0.58–0.93)	0.01	0.56	(0.43–0.72)	< 0.001
Gender							
Female	123	1	reference		1	reference	
Male	155	1.55	(1.23–1.97)	< 0.001	1.71	(1.35–2.18)	< 0.001
Age, years							
< 40	2	1	reference		1	reference	
40–59	58	5.83	(1.42–23.87)	0.01	5.42	(1.32–22.25)	0.02
60–79	192	15.63	(3.88–63.02)	< 0.001	13.45	(3.31–54.59)	< 0.001
≥ 80	26	14.79	(3.5–62.56)	< 0.001	12.24	(2.87–52.18)	< 0.001
Baseline comorbidity							
Hypertension							
No	70	1	reference		1	reference	
Yes	208	1.11	(0.85–1.46)	0.45	1.33	(0.99–1.79)	0.06
Diabetes mellitus							
No	169	1	reference		1	reference	
Yes	109	0.84	(0.66–1.06)	0.15	0.81	(0.63–1.05)	0.11
Moderate or severe liver disease							
No	275	1	reference		1	reference	
Yes	3	0.77	(0.25–2.39)	0.65	0.83	(0.26–2.59)	0.75
Myocardial infarction							
No	269	1	reference		1	reference	
Yes	9	0.53	(0.27–1.03)	0.06	0.59	(0.3–1.15)	0.12
Congestive heart failure							
No	230	1	reference		1	reference	
Yes	48	0.84	(0.61–1.15)	0.27	0.87	(0.63–1.2)	0.39
Ischemic stroke							
No	126	1	reference		1	reference	
Yes	152	0.86	(0.68–1.09)	0.21	0.83	(0.64–1.07)	0.15
Drugs							
Anti-hypertensive agents							
No	75	1	reference		1	reference	
Yes	203	1.92	(1.47–2.5)	< 0.001	1.94	(1.45–2.6)	< 0.001
Hypoglycemic agents							
No	265	1	reference		1	reference	
Yes	13	0.88	(0.51–1.54)	0.67	0.85	(0.48–1.48)	0.56
Coumadin and heparin							
No	271	1	reference		1	reference	
Yes	7	0.61	(0.29–1.28)	0.19	0.52	(0.24–1.11)	0.09
Other antithrombotic agents							
No	232	1	reference		1	reference	
Yes	46	1.18	(0.86–1.62)	0.30	1.06	(0.76–1.48)	0.72
NSAIDs							
No	134	1	reference		1	reference	
Yes	144	0.83	(0.66–1.05)	0.13	0.83	(0.65–1.05)	0.12

Abbreviations: *HR* Hazard ratio, *CI* Confidence interval, *NSAIDs* Non-steroidal anti-inflammatory drugs

Adjusted HR: adjusted for gender, age, hypertension, diabetes mellitus, moderate or severe liver disease, myocardial infarction, congestive heart failure, ischemic stroke, anti-hypertension agents, hypoglycemic agents, coumadin and heparin, other antithrombotic agents and NSAIDs in Cox proportional hazards regression

Table 3 shows that aspirin use significantly decreased the risk of HCC in both genders (female: adjusted HR = 0.51, 95% CI = 0.35–0.76, *p < 0.001*; male: adjusted HR = 0.59, 95% CI = 0.42–0.83, *p < 0.01*). However, with respect to patients in the same age strata, only the aspirin users who were 60 to 79 years old showed a significant decrease in HCC risk compared to the non-aspirin users (adjusted HR = 0.56, 95% CI = 0.41–0.76, *p < 0.001*). Among the anti-hypertensive agent users, meanwhile aspirin also had an apparent benefit of decreased HCC risk (adjusted HR = 0.53, 95% CI = 0.4–0.71, *p < 0.001*).

**Table 3:** Incidence rates, hazard ratios, and confidence intervals of HCC among different stratifications of HCV patients with and without aspirin use

Variables	Aspirin						Aspirin VS. Non-aspirin	
	No (*n* = 1911)			Yes (*n* = 1911)			Crude HR (95% CI)	Adjusted HR (95% CI)
	Event	Person years	IR^†^	Event	Person years	IR^†^		
Overall	147	6848	2.15	131	8546	1.53	0.73(0.58–0.93)**	0.56(0.43–0.72)***
Gender								
Female	66	3838	1.72	57	4707	1.21	0.72(0.51–1.03)	0.51(0.35–0.76)***
Male	81	3010	2.69	74	3839	1.93	0.72(0.53–0.99)*	0.59(0.42–0.83)**
Age, years								
< 40	0	592	0	2	633	0.32		
40–59	31	2929	1.06	27	3069	0.88	0.83(0.5–1.39)	0.64(0.35–1.14)
60–79	98	2888	3.39	94	4325	2.17	0.65(0.49–0.87)**	0.56(0.41–0.76)***
≥ 80	18	440	4.09	8	518	1.54	0.44(0.19–1.01)	0.45(0.18–1.11)
Baseline comorbidity								
Hypertension								
No	39	1713	2.28	31	2557	1.21	0.55(0.34–0.88)*	0.39(0.23–0.65)***
Yes	108	5135	2.1	100	5989	1.67	0.81(0.62–1.06)	0.63(0.47–0.85)**
Diabetes mellitus								
No	90	3692	2.44	79	5060	1.56	0.65(0.48–0.88)**	0.49(0.35–0.68)***
Yes	57	3156	1.81	52	3486	1.49	0.85(0.58–1.24)	0.68(0.45–1.03)
Moderate or severe liver disease								
No	145	6711	2.16	130	8468	1.54	0.73(0.57–0.92)**	0.56(0.43–0.73)***
Yes	2	137	1.46	1	78	1.28	0.8(0.07–8.85)	
Myocardial infarction								
No	143	6443	2.22	126	8057	1.56	0.72(0.57–0.92)**	0.55(0.42–0.72)***
Yes	4	405	0.99	5	489	1.02	1.02(0.27–3.82)	0.72(0.16–3.23)
Congestive heart failure								
No	127	5443	2.33	103	6969	1.48	0.65(0.5–0.84)**	0.52(0.39–0.69)***
Yes	20	1406	1.42	28	1577	1.78	1.25(0.7–2.23)	0.79(0.42–1.5)
Ischemic stroke								
No	66	2754	2.4	60	3858	1.56	0.66(0.46–0.93)*	0.48(0.33–0.71)***
Yes	81	4094	1.98	71	4688	1.51	0.79(0.58–1.09)	0.62(0.43–0.88)**
Drugs								
Anti-hypertensive agents								
No	56	4333	1.29	19	2189	0.87	0.69(0.41–1.16)	0.58(0.33–1.01)
Yes	91	2515	3.62	112	6357	1.76	0.51(0.38–0.67)***	0.53(0.4–0.71)***
Hypoglycemic agents								
No	141	6548	2.15	124	8056	1.54	0.73(0.57–0.93)*	0.55(0.42–0.72)***
Yes	6	300	2	7	490	1.43	0.65(0.21–2.03)	0.71(0.2–2.52)
Coumadin and heparin								
No	145	6765	2.14	126	8037	1.57	0.75(0.59–0.95)*	0.56(0.43–0.73)***
Yes	2	83	2.4	5	509	0.98	0.4(0.07–2.19)	0.41(0.05–3.18)
Other antithrombotic agents								
No	136	6531	2.08	96	6703	1.43	0.71(0.54–0.92)**	0.53(0.4–0.7)***
Yes	11	318	3.46	35	1843	1.9	0.56(0.29–1.12)	0.68(0.34–1.37)
NSAIDs								
No	69	3535	1.95	65	3108	2.09	1.07(0.76–1.5)	0.79(0.54–1.14)
Yes	78	3313	2.35	66	5437	1.21	0.54(0.39–0.74)***	0.41(0.29–0.59)***

Abbreviations: *IR†* Incidence rates, per 100 person-years, *HR* Hazard ratio, *CI* Confidence interval, *NSAIDs* Non-steroidal anti-inflammatory drugs

* *p* < 0.05, ** *p* < 0.01, *** *p* < 0.001

Adjusted HR: adjusted for gender, age, hypertension, diabetes mellitus, moderate or severe liver disease, myocardial infarction, congestive heart failure, ischemic stroke, anti-hypertension agents, hypoglycemic agents, coumadin and heparin, other antithrombotic agents and NSAIDs in Cox proportional hazards regression

For the aspirin users, we classified the duration of aspirin use into four levels (< 1 year, 1–2 years, 2–3 years, ≥3 years) while also classifying the non-users according to the same four levels for reference. Among the aspirin users, the most events occurred among those whose duration of aspirin use was less than 1 year (Table 4). Compared with the non-use of aspirin, all of the different durations of aspirin use had significant effects in terms of reducing HCC risk, except the duration of 2–3 years (adjusted HR = 0.6, 95% CI = 0.32–1.13, *p = 0.11*). The lowest adjusted HR was 0.33 (95% CI = 0.18–0.61, *p < 0.001*), which was found for the aspirin users with a duration of aspirin use of 1–2 years.

**Table 4:** The dose responses to aspirin among the HCV patients

Duration of aspirin use	Event no.	Crude			Adjusted		
	(*n* = 278)	HR	(95% CI)	*p* value	HR	(95% CI)	*p* value
Non-user	147	1	reference		1	reference	
< 1 year	93	0.74	(0.57–0.96)	0.02	0.63	(0.48–0.83)	< 0.001
1–2 years	12	0.52	(0.29–0.93)	0.03	0.33	(0.18–0.61)	< 0.001
2–3 years	11	0.94	(0.51–1.73)	0.84	0.6	(0.32–1.13)	0.11
≥3 years	15	0.8	(0.47–1.37)	0.42	0.45	(0.26–0.79)	0.005

Abbreviations: *HR* Hazard ratio, *CI* Confidence interval, *NSAIDs* Non-steroidal anti-inflammatory drugs

Adjusted HR: adjusted for gender, age, hypertension, diabetes mellitus, moderate or severe liver disease, myocardial infarction, congestive heart failure, ischemic stroke, anti-hypertension agents, hypoglycemic agents, coumadin and heparin, other antithrombotic agents and NSAIDs in Cox proportional hazards regression

## Discussion

Using a nationwide population database, we investigated the association between the use of aspirin and HCC risk in HCV carriers. This is the first study to find that HCV carriers who used aspirin had a lower risk of HCC than HCV carriers who did not use aspirin (adjusted HR = 0.56, 95% CI = 0.43–0.72, *p < 0.001*; Table 2). Furthermore, the male HCV carriers had a 1.71-fold higher risk of HCC than the female HCV carriers (adjusted HR = 1.71, 95% CI = 1.35–2.18, *p < 0.001;* Table 2), although aspirin treatment significantly reduced the HCC risk in both genders (female: adjusted HR = 0.51, 95% CI = 0.35–0.76, *p < 0.001*; male: adjusted HR = 0.59, 95% CI = 0.42–0.83, *p < 0.01*; Table 3). The Kaplan-Meier curves showed that the HCV carriers treated with aspirin had a lower cumulative incidence rate of HCC than those not treated with aspirin (*p < 0.0001*; Fig. 2). In considering the duration of aspirin use, the lowest adjusted HR for developing HCC was 0.33 (95% CI = 0.18–0.61, *p < 0.001*), which was found in those aspirin users with a duration of aspirin use of 1–2 years. Overall, the results of our study demonstrate that aspirin treatment can decrease the occurrence of HCC in HCV carriers.

Taiwan is one of the HBV endemic areas and has a substantial proportion of patients with dual HBV and HCV infection [22]. To prevent interference from HBV infections, we excluded HBV carriers in order to purify our study group. Since interferon or direct-acting anti-viral treatment might totally cure an HCV infection [23], our study also excluded those patients who received interferon or direct-acting anti-viral treatment before the index date. This guaranteed that the investigated patients were HCV carriers while also ensuring that any lowering of the HCC occurrence was due only to the use of aspirin.

The HCC risk in the individuals with the comorbidity of hypertension was similar to that in those without hypertension. As shown in Table 2, the comorbidity of hypertension was not associated with an increased occurrence of HCC in our study population. It is interesting, however, to note that the HCC risk among participants receiving anti-hypertensive agents was 1.94-fold higher than that among those not receiving such agents (95% CI = 1.45–2.6, *p < 0.001;* Table 2). Although our present findings cannot explain this conflict, Ho et al. [24] found that the use of angiotensin-converting enzyme inhibitors and angiotensin II receptor blockers was associated with higher HCC occurrence in patient subgroups consisting of patients with no cirrhosis, no DM, and no hyperlipidemia. This issue requires further study in the future.

The real mechanism by which aspirin reduces HCC risk in HCV carriers is not well understood. A previous study, however, revealed different pathways for the viral and non-viral carcinogenesis of HCC. In an HBV transgenic mice model, it was found that platelets facilitate immune-mediated liver damage through the accumulation of HBV-specific cytotoxic T lymphocytes (CTLs) [25]. Sitia et al. [26] further found that aspirin decreased T-cell mediated inflammation, liver fibrosis, and progression to HCC in this HBV transgenic mice model. Whether the HBV-related carcinogenesis of HCC and the effect of aspirin on immunomodulation are the same as in HCV is still under investigation. However, aspirin has been found to have antioxidative and antiviral activity in HCV-expressing cells through Cu/Zn superoxide dismutase (SOD1) induction [27] and the downregulation of inducible nitric oxide synthase (iNOS) [28]. Moreover, Trujillo-Murillo et al. [29] found that acetylsalicylic acid decreases HCV replication via the inhibition of COX-2 expression through the activation of p38 and mitogen-activated protein kinase/extracellular signal-regulated kinase kinase 1/2 (MEK1/2). In a more recent study, Yin and Zhang [30] investigated the effects of aspirin on the blocking of HCV entry. They discovered that aspirin degrades claudin-1, an HCV receptor, through the proteasome degradation pathway and inhibits the entry of all genotypes of HCV pseudoparticles [30]. Taken together, past studies suggest that aspirin could reduce HCV expression and prevent HCV re-infection in patients with chronic HCV infection through COX-2-dependent and COX-2-independent pathways [19].

We found that aspirin lowered the HCC incidence rate in HCV carriers over the first 10 years of aspirin treatment, including a 67% reduction in the occurrence rate in cases in which the duration of aspirin treatment was less than one year (Table 4; Fig. 2). Moreover, the cumulative HCC incidence rate became even lower over time. Our hypothesis is that aspirin could relieve chronic inflammation via the inhibition of the cyclooxygenase enzyme, thus lowering the occurrence of HCC at first [31, 32]. However, the inhibition of the cyclooxygenase enzyme also restrains the immune system, such that an HCV infection may become uncontrolled [33, 34]. Due to the resulting accumulation of liver cell damage and the duplication of HCV, the HCC incidence rate then gradually becomes higher. Nonetheless, regular close follow-up visits and aspirin treatment might help lower the incidence rate of HCC in HCV carriers, especially over the first 10 years of aspirin treatment. That said, further clinical trials are warranted to clarify the preventive effects of aspirin against HCC risk in HCV carriers.

The present study, which was based on data from National Health Insurance program in Taiwan, had the following advantages: First, the large national sample population can be taken as representative of the entire population of Taiwan from 2000 to 2013; nearly all patients in Taiwan could get the proper medical care; and only a few people would take over-the-counter drugs themselves. Hence, we could retrieve the details of patients’ information and medical records from the NHIRD. Second, cancers are categorized as serious diseases in Taiwan, such that cancer patients can apply for a “catastrophic illness card” as part of the NHI program. Most such patients’ medical visits are free when they visit outpatient departments or are admitted to a hospital, and almost all cancer patients will receive medical treatment. The present study, therefore, could identify HCV carriers and HCC patients accurately. Third, we could rather precisely collect information for 16,466 HCC patients to perform a 1:1 propensity score matching by age, sex, comorbidities, drugs, treatment course of aspirin, and index year. Due to the gross sample size and detailed/accurate matching, we could then precisely analyze the association of aspirin use with HCC risk in the HCV carriers.

There were some limitations to this study. First, the study was a retrospective study. As such, we could not trace the HCV titer in these patients and thus could not prove our hypothesis that the HCV titer might flare up because of the restrained immune response owing to aspirin. Second, there were no data regarding the aspirin doses per day of the patients, because definite data in that regard were not included in the database. However, the prescribed dose in Taiwan is usually 100 mg per day. Third, alcohol consumption also leads to a higher incidence of HCC among HCV-infected individuals. However, the effects of alcohol consumption could not be measured in this retrospective study.

## Conclusion

In conclusion, the use of aspirin appeared to reduce the risk of HCC in HCV carriers, with the hazard ratio of 0.56, (95% CI = 0.43–0.72, *p < 0.001*). Moreover, both genders exhibited significant reductions in the occurrence of HCC after aspirin use. Further clinical trials are warranted to clarify the preventive effects of aspirin against HCC risk in HCV carriers.

## Data Availability

The datasets used and analyzed during the current study are available from the corresponding author on reasonable request.

## Abbreviations

aHR: Adjusted hazard ratio
CIs: Confidence intervals
COX-2: Cyclooxygenase-2
HBV: Hepatitis B virus
HCC: Hepatocellular carcinoma
HCV: Hepatitis C virus
NHI: National Health Insurance
NHIRD: National Health Insurance Research Database
NSAIDs: Non-steroidal anti-inflammatory drugs
